# The association between body mass index and total lumbar bone mineral density in obese adults: the national health and nutrition examination survey (NHANES) 2011–2020

**DOI:** 10.1007/s42000-025-00699-3

**Published:** 2025-07-24

**Authors:** Chao Su, Peng Wang, Dandan Lian, Chen Sun, Cunchuan Wang

**Affiliations:** 1 Department of Gastrointestinal Surgery, Cheeloo College of Medicine, Weihai Municipal Hospital, Shandong University, Weihai, 264200 China; 2 Department of Metabolic and Bariatric Surgery, The First Affiliated Hospital of Jinan University, No.601, West Huangpu Avenue, Tianhe District, Guangzhou, 510632 China; 3 Department of Spine Surgery, Cheeloo College of Medicine, Weihai Municipal Hospital, Shandong University, Weihai, 264200 China; 4 Department of Pediatric Surgery, Cheeloo College of Medicine, Weihai Municipal Hospital, Shandong University, Weihai, 264200 China

**Keywords:** Obesity, Body mass index, BMD, Osteoporosis, NHANES

## Abstract

**Introduction:**

This study aimed to investigate the relationship between BMI and total lumbar BMD in obese adults.

**Methods:**

The current cross-sectional study included 3,708 obese individuals. The data on BMI, total lumbar BMD, and other covariates were obtained from the National Health and Nutritional Examination Survey (NHANES) (http://www.cdc.gov/nchs/nhanes/) between 2011 and March 2020 prior to the COVID-19 pandemic. Multivariate logistic regression models were utilized to investigate the association between BMI and total lumbar BMD. Smooth curve fittings and generalized additive models were used to analyze the potential non-linearity.

**Results:**

A total of 3,708 participants (1,610 males and 2,098 females) were included in the study. In multivariate regression analysis, the association between BMI and total lumbar BMD was positive (β = 0.003, 95% CI: 0.002, 0.004). This relationship still existed after being adjusted for gender, age, and race (β = 0.003, 95% CI: 0.002, 0.004) and fully adjusted for all covariates (β = 0.003, 95% CI: 0.002, 0.004). In threshold effect analysis, the relationship between BMI and total lumbar BMD followed a U-shaped curve, with the inflection point at 36.1 kg/m^2^.

**Conclusion:**

The present study revealed a positive association between BMI and lumbar BMD in obese adults, the association notably following a U-shaped curve with an inflection point at a BMI of 36.1 kg/m².

**Supplementary Information:**

The online version contains supplementary material available at 10.1007/s42000-025-00699-3.

## Introduction

Obesity and osteoporosis have become major global health problems in recent decades with their prevalence steadily increasing. Obesity, as defined by a body mass index (BMI) greater than 30 kg/m^2^, can lead to a series of metabolic diseases [1, 2]: it constitutes the fifth leading cause of overall mortality, accounting for at least 2.8 million adult deaths each year [3]. Osteoporosis is a common metabolic skeletal disease characterized by reduced bone mineral density (BMD) and progressive microarchitectural deterioration, resulting in increased risk of fragility fractures and subsequent disability and affecting more than 200 million people globally with high healthcare costs being incurred [4].

Over the last few years, several studies have attempted to explain the complex association between obesity and osteoporosis. Obesity was once assumed to protect against osteoporosis but multiple studies are now challenging this hypothesis [5]. The interaction between obesity and osteoporosis is complex and not fully understood. The increase in the prevalence of both conditions prompts the need to better understand the association between obesity and osteoporosis. Therefore, the aim of the current study was to evaluate the relationship between BMI and total lumbar BMD in obese adults aged over 17 years using a nationally representative sample from the National Health and Nutritional Examination Survey (NHANES) 2011–2020 (http://www.cdc.gov/nchs/nhanes/).

## Materials and methods

### Study population

This study analyzed data from the NHANES between 2011 and March 2020 prior to the coronavirus disease 2019 pandemic. NHANES databases are cross-sectional surveys providing vast amounts of information concerning the health and nutritional status of the general United States population.

NHANES is conducted by the National Center for Health Statistics and approved by the NCHS Ethics Review Board [6]. All participants signed written informed consent forms. The current study was approved by our institutional Ethics Committee. All NHANES data are publicly available online. More detailed information about the survey can be obtained from the NHANES website.

**Fig. 1:**
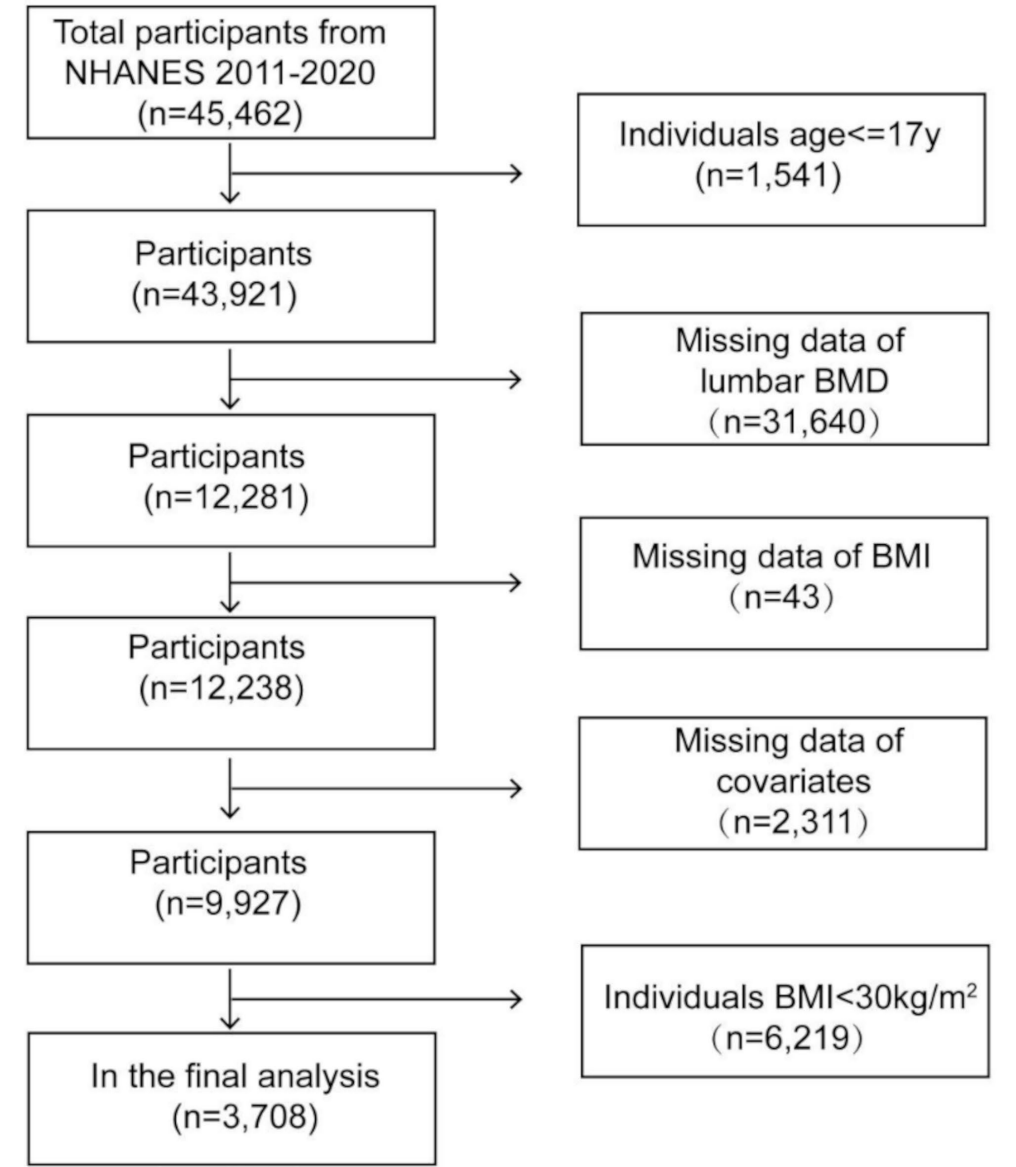
Flow chart of sample selection from NHANES 2011–2020

The NHANES data for 2011–2020 were combined for the present study. The study population was restricted to obese adults with a BMI greater than 30 kg/m^2^. Among the 43,921 adults aged over 17 years, we excluded 31,640 participants with missing total lumbar BMD data, 43 participants with missing BMI data, 2,311 participants with missing covariates data, and 6,219 participants with a BMI of less than 30 kg/m^2^. Finally, a total of 3,708 participants were analyzed after applying these exclusion criteria (Fig. 1).

### Study variables

BMI (calculated as weight in kilograms divided by height in meters squared, rounded to one decimal place) was the exposure variable. The outcome variable was total lumbar BMD. Total lumbar BMD (measured by DXA scans) was identified as the outcome variable, its administration being carried out by certified and trained radiology technologists. The spine scans were acquired using Hologic Discovery model A densitometers (Hologic, Inc., Bedford, MA, USA) in 2011–2018 and using Hologic Horizon model A densitometers (Hologic, Inc., Bedford, MA, USA) in 2019 until March 2020. All scans were analyzed with Hologic APEX version 4.0 software. From 2019 to March 2020, three new DXA densitometer systems were installed in the NHANES mobile examination center. A cross-calibration study was conducted to assure the accuracy of the NHANES longitudinal assessment.

Multivariate models contain variables that might confound the links between BMI and total lumbar BMD. Data on gender, age, race, and education level were obtained from questionnaires. The data on albumin (ALB, g/L), alanine aminotransferase (ALT, U/L), aspartate aminotransferase (AST, U/L), alkaline phosphatase (ALP, IU/L), blood urea nitrogen (BUN, mmol/L), calcium (Ca, mmol/L), creatine phosphokinase (CK, U/L), creatinine (Cr, µmol/L), gammaglutamyl transaminase (GGT, IU/L), glucose (Glu, mmol/L), iron (Fe, µmol/L), phosphorus (P, mmol/L), bilirubin (TBIL, µmol/L), total protein (TP, g/L), uricacid (UA, µmol/L), sodium (Na+, mmol/L), potassium (K+, mmol/L), chloride (Cl-, mmol/L), osmolality, and globulin (GLO, g/L) serum levels were obtained from standard biochemistry profile. Detailed information about these covariates can be obtained from the NHANES website.

### Statistical analyses

The study participants were stratified into tertiles according to obesity classification defined by BMI. Categorical variables were expressed as frequencies (percentages). Comparisons among groups were conducted using the chi-square test. Continuous variables were presented as mean ± standard deviation (SD). Evaluation was carried out using the t-test. Weighted multivariable linear regression models were applied to analyze the association between BMI and total lumbar BMD. Weighted smooth curve fittings and generalized additive models were used to characterize the potential non-linearity. Two-piecewise linear regression models were established to calculate the threshold effects if non-linearity associations existed. Three models were built, as follows: Model I, an unadjusted model; Model II, minimally adjusted for gender, age, and race; and Model III, fully adjusted for all covariates (listed in Tables 1 and 2). All analyses were performed using Empower-Stats software (version 2.0. X&Y Solutions, Boston, MA, USA) and statistical software R (version 3.4.3). The p-value < 0.001 was considered statistically significant.

**Table 1:** Weighted characteristics of the study population based on BMI levels tertiles

BMI	Q1	Q2	Q3	*P* -value
Age	46.27 ± 13.66	45.68 ± 13.87	44.67 ± 12.75	0.0269
Gender				< 0.0001
Male	54.12	43.32	35.20	
Female	45.88	56.68	64.80	
Race				< 0.0001
1	63.12	60.18	57.90	
2	12.49	16.40	20.84	
3	11.44	11.81	11.04	
4	12.95	11.61	10.23	
EL				0.3439
1	38.37	36.67	35.00	
2	60.19	61.35	62.82	
AL (g/L)	42.81 ± 3.18	41.78 ± 3.24	40.53 ± 3.24	< 0.0001
ALT (U/L)	29.74 ± 19.82	29.10 ± 20.72	29.33 ± 20.73	0.7002
AST (U/L)	26.42 ± 13.92	25.51 ± 22.30	25.80 ± 13.95	0.3299
ALP (IU/L)	70.03 ± 23.95	72.67 ± 22.61	75.84 ± 28.34	< 0.0001
BUN (mmol/L)	4.79 ± 1.61	4.79 ± 1.94	4.69 ± 2.13	0.4436
CAL (mmol/L)	2.34 ± 0.08	2.33 ± 0.09	2.32 ± 0.09	< 0.0001
CPK (IU/L)	158.25 ± 146.83	151.53 ± 128.56	153.51 ± 171.64	0.4668
CR (umol/L)	77.25 ± 24.69	75.97 ± 30.37	74.91 ± 31.78	0.1261
GGT (IU/L)	34.71 ± 44.66	31.75 ± 43.55	30.55 ± 31.37	0.0402
GLU (mmol/L)	5.69 ± 2.05	6.09 ± 2.44	6.25 ± 2.49	< 0.0001
IR (umol/L)	15.24 ± 6.25	13.53 ± 5.18	12.28 ± 5.54	< 0.0001
PHO (mmol/L)	1.19 ± 0.17	1.16 ± 0.18	1.19 ± 0.18	< 0.0001
BIL (umol/L)	10.19 ± 4.80	9.39 ± 5.50	8.72 ± 4.95	< 0.0001
PRO (g/L)	71.05 ± 4.30	70.64 ± 4.54	70.84 ± 4.42	0.0467
UA (mg/dL)	5.66 ± 1.34	5.79 ± 1.39	6.01 ± 1.46	< 0.0001
SOD (mmol/L)	139.11 ± 2.31	139.07 ± 2.31	138.88 ± 2.51	0.0808
POT (mmol/L)	3.98 ± 0.32	3.97 ± 0.32	4.01 ± 0.34	0.0186
CL (mmol/L)	103.66 ± 2.92	103.64 ± 3.06	103.52 ± 3.08	0.5603
OSM (mmol/Kg)	278.06 ± 4.96	278.37 ± 5.27	278.05 ± 5.19	0.2536
GLO (g/L)	28.25 ± 4.10	28.85 ± 4.41	30.31 ± 4.17	< 0.0001
BMD (gm/cm2)	1.04 ± 0.15	1.04 ± 0.16	1.09 ± 0.16	< 0.0001

Notes: Mean + SD for contimuous variable; % for categorical variable; *P* value was calculated using the weighted chi-square test

The tertiles (Q1–Q3) were defined as: Q1: 30–34.9 kg/m², Q2: 35–39.9 kg/m², Q3: ≥40 kg/m²

**Table 2:** Associations between BMI and total spine BMD (gm/cm^2^)

	Model I β (95% CI) *P*	Model II β (95% CI) *P*	Model III β (95% CI) *P*
BMI	0.003 (0.002, 0.004) < 0.00001	0.003 (0.002, 0.004) < 0.00001	0.003 (0.002, 0.004) < 0.00001
Stratified by BMI (tertiles)			
Q1	Reference	Reference	Reference
Q2	-0.004 (-0.015, 0.008) 0.55682	-0.005 (-0.016, 0.007) 0.44727	-0.008 (-0.019, 0.004) 0.19064
Q3	0.050 (0.036, 0.063) < 0.00001	0.046 (0.033, 0.060) < 0.00001	0.042 (0.028, 0.056) < 0.00001
*P* for trend	< 0.001	< 0.001	< 0.001
Stratified by gender			
Males	0.005 (0.003, 0.006) < 0.00001	0.004 (0.003, 0.006) < 0.00001	0.004 (0.003, 0.006) < 0.00001
Females	0.003 (0.002, 0.004) < 0.00001	0.002 (0.001, 0.003) 0.00002	0.002 (0.001, 0.003) 0.00017
Stratified by race			
White	0.003 (0.001, 0.004) 0.00159	0.003 (0.001, 0.005) 0.00062	0.003 (0.001, 0.004) 0.00379
Black	0.003 (0.001, 0.005) 0.00025	0.003 (0.002, 0.005) 0.00003	0.003 (0.001, 0.005) 0.00049
Mexican	0.004 (0.002, 0.006) 0.00010	0.004 (0.002, 0.006) 0.00004	0.004 (0.002, 0.007) 0.00003
Other	0.003 (0.001, 0.005) 0.00162	0.003 (0.001, 0.005) 0.00102	0.003 (0.001, 0.005) 0.00895

Model I: No covariates were adjusted; Model II: gender (not adjusted for in the subgroup analyses), age, and race (not adjusted for in the subgroup analyses) were adjusted; Model III: gender (not adjusted for in the subgroup analyses), age, race (not adjusted for in the subgroup analyses), education level, albumin, alanine aminotransferase, aspartate aminotransferase, alkaline phosphatase, blood urea nitrogen, calcium, creatine phosphokinase, creatinine, gammaglutamyl transaminase, glucose, iron, phosphorus, bilirubin, total protein, uric acid, sodium, potassium, chloride, osmolality, and globulin

Abbreviations: BMI, body mass index; BMD, Total spine BMD

## Results

A total of 1,610 men and 2,098 women are included in this study. Baseline characteristics of all subjects, classified by tertiles of BMI levels, are respectively presented in Table 1. As shown in Table 1, compared to the Q3 group, participants with lower BMI levels were older and had a lower lumbar BMD.

The association between BMI and total lumbar BMD was positive in all three regression models (Table 2), as follows: model I (β = 0.003, 95% CI: 0.002, 0.004), model II (β = 0.003, 95% CI: 0.002, 0.004), and model III (β = 0.003, 95% CI: 0.002, 0.004), with this relationship being more prominent in the Q3 group. In the subgroup analysis stratified by gender and race, we also observed a positive association between BMI and total lumbar BMD after controlling for potential confounding factors, with all p-values < 0.001.

**Fig. 2:**
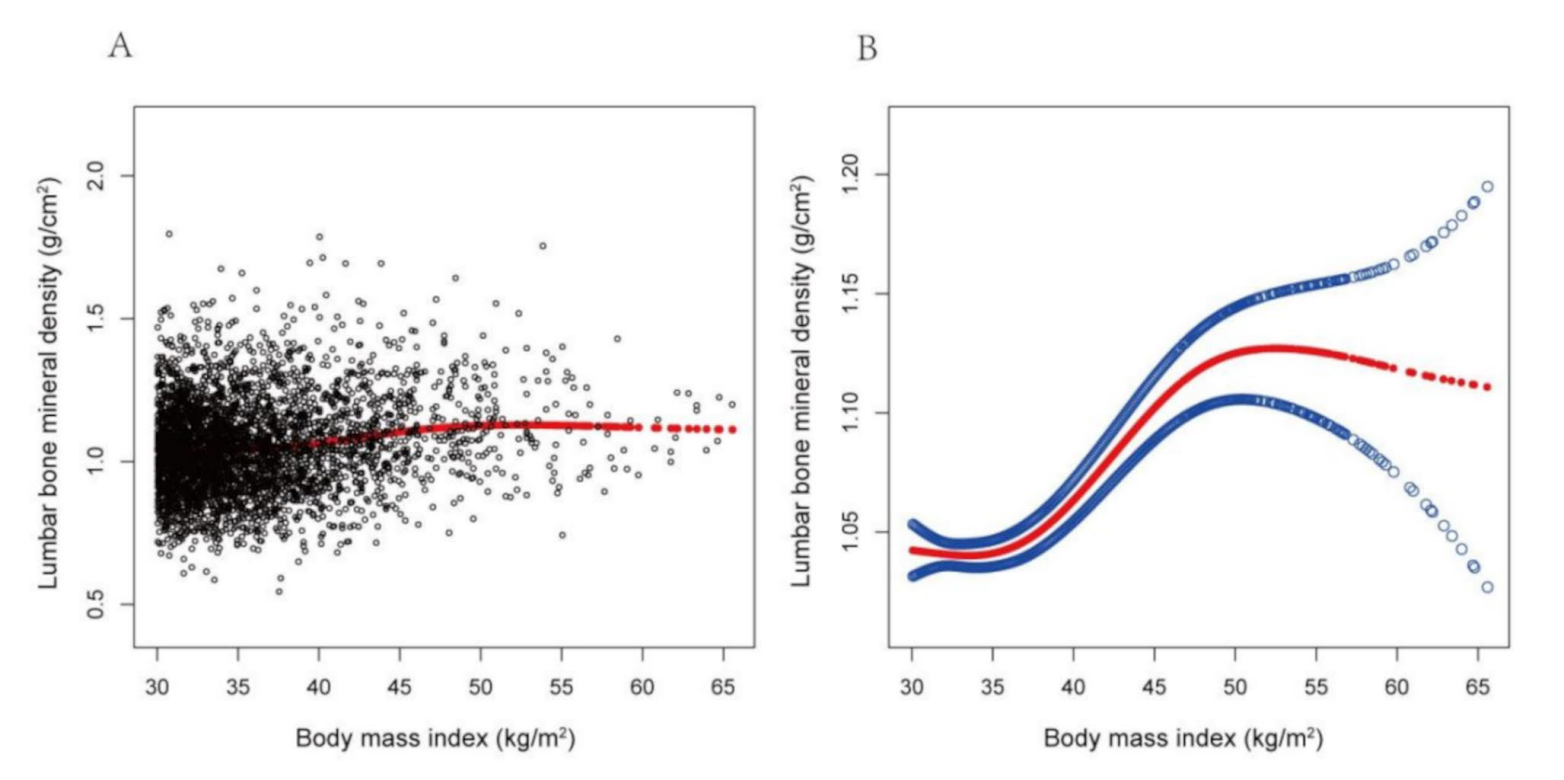
The association between body mass index (BMI) and lumbar bone mineral density (BMD) (**A**) Each black point represents a single participant BMI sample. (**B**) The solid red line represents the smooth curve fit between variables. Blue bands represent the 95% of confidence interval from the fit. Gender, age, race, marital status, alanine aminotransferase, albumin, alkaline phosphatase, bicarbonate, blood urea nitrogen, chloride, creatinine, globulin, glucose, gamma glutamyl transferase, iron, osmolality, phosphorus, sodium, total bilirubin, total calcium, cholesterol, total protein, and uric acid were adjusted

The non-linear relationship between BMI and total lumbar BMD is shown in Fig. 2. Using a two-piecewise linear regression model, the point of inflection in the U-shaped association was at a level of 36.1 for BMI. To the left of the inflection point (BMI < 36.1 kg/m²), increasing BMI was associated with decreasing lumbar BMD. To the right (BMI ≥ 36.1 kg/m²), higher BMI correlated with increased BMD, and the difference was statistically significant (Table 3).

**Table 3:** Threshold effect analysis of BMI on total lumbar BMD using the two-piecewise linear regression model

Total lumbar BMD	Adjusted β (95% CI) *P*
Fitting by the standard linear model	0.003 (0.002, 0.004) < 0.0001
Fitting by the two-piecewise linear model	
Inflection point	36.1
BMI < 36.1 ( kg/m^2^)	-0.003 (-0.006, -0.001) 0.0187
BMI > 36.1 ( kg/m^2^)	0.006 (0.004, 0.007) < 0.0001
Log likelihood ratio	< 0.001

Notes: gender, age, race, education level, albumin, alanine aminotransferase, aspartate aminotransferase, alkaline phosphatase, blood urea nitrogen, calcium, creatine phosphokinase, creatinine, gammaglutamyl transaminase, glucose, iron, phosphorus, bilirubin, total protein, uric acid, sodium, potassium, chloride, osmolality, and globulin were adjusted

Abbreviations: BMI, body mass index; BMD, total spine BMD

Table 3 focuses on analyzing the relationship between BMI and total lumbar BMD. BMD, especially in the lumbar spine, is an important indicator for assessment of bone health and fracture risk. The standard linear model indicated a positive correlation between BMI and lumbar BMD (β = 0.003, *p* < 0.0001). Specifically, for each additional BMI unit, the average increase in lumbar BMD is 0.003, this relationship being statistically significant (*P* < 0.0001). This means that, at least in this dataset, there is a positive correlation between higher BMI and higher lumbar BMD.

Compared to the standard linear model, the two-stage linear model provides a more complex perspective to examine the relationship between BMI and BMD. This model suggests that the relationship between BMI and BMD may change before and after reaching a specific inflection point.

The turning point here is BMI = 36.1. This indicates that there may be significant differences in the relationship between BMI and BMD before and after reaching this value.

Within the range of BMI below 36.1, BMI shows a marginally inverse association with BMD, with an average decrease of 0.003 (*P* = 0.0187) for each additional BMI unit. This contrasts sharply with the observed results of the standard linear model.

However, when the BMI exceeds 36.1, its relationship with BMD becomes positively correlated again, with an increase of 0.006 (*P* < 0.0001) for each additional BMI unit.

This bilinear relationship indicates that reliance solely on a linear model may not accurately capture the true relationship between BMI and BMD. The two-stage linear model provides a more complex but possibly more realistic description.

## Discussion

The association between BMI and lumbar BMD has been extensively investigated across diverse demographic cohorts. However, the specific relationship is nonlinear and context-dependent, with studies reporting divergent conclusions. Notably, a significant positive association between BMI and lumbar BMD with the coeffietient 0.056 has been documented in Tang’s study [7]. While the biological mechanisms underlying this association remain only partially elucidated, two prominent hypotheses have emerged. The mechanical loading hypothesis posits that chronically elevated mechanical stress on weight-bearing bones in individuals with higher BMI induces osteogenic activity, thereby augmenting BMD [7]. Alternatively, an endocrine-mediated pathway has been proposed involving regulatory interplay between adipokines (insulin-like growth factor 1 [8]) and bone-active hormones (estrogen [9]), which modulate the homeostasis of adipose and osseous tissues.

In addition to the positive correlation between BMI and BMD, negative correlations have also been found in other studies, with several different mechanisms being reported to be involved in those negative relationships. For example, obesity-related oxidative stress and low-grade chronic inflammation may stimulate the activity of osteoclasts, leading to reduced BMD [10, 11]. Obesity could also stimulate pre-osteoblasts to differentiate toward adipocytes rather than osteoblasts, resulting in increased bone fragility [12].

Our present study showed a positive association between BMI and lumbar BMD. In the subgroup analysis stratified by gender, this positive correlation maintains consistency, though some disparity was observed between genders. Although the relationship between female BMI and BMD is significant in all three models, its correlation is slightly lower than that of males. This may be related to biological differences in women, hormone levels, or health behavior.

The observed sexual dimorphism in body composition and endocrine signaling likely arises from divergent adipose tissue distribution patterns and hormonal regulation [13]. Women exhibit estrogen dominance in bone metabolism, whereas men rely on testosterone’s direct effects and its indirect contribution via peripheral aromatization to estrogen [14]. Males demonstrate preferential accumulation of visceral adipose tissue (VAT) [15], a metabolically active depot strongly associated with chronic low-grade systemic inflammation. VAT functions as an endocrine organ, releasing proinflammatory cytokines (e.g., TNF-α and IL-6) and free fatty acids that mechanistically contribute to insulin resistance and oxidative stress through dysregulation of glucose homeostasis and mitochondrial dysfunction [16]. These factors inhibit osteoblast activity while stimulating osteoclastogenesis, partially offsetting the mechanical benefits of higher body weight on bone [17]. Additionally, males exhibit lower circulating estrogen levels compared to females, reducing the antiresorptive effects of estrogen on bone metabolism [9]. This dual burden of inflammation and diminished hormonal protection may explain the stronger positive correlation between BMI and BMD in males, as mechanical loading alone cannot fully counteract metabolic harms. Conversely, females have higher subcutaneous fat [13], which may secrete adipokines (leptin), with mixed effects on bone metabolism [18, 19]. Estrogen’s protective role in bone health [20] might further explain the weaker correlation in females.

The observed racial disparities in the association between BMI and lumbar BMD may stem from multifactorial interactions involving genetic, hormonal, and body composition differences. For instance, African American populations are reported to have higher baseline BMD compared to Caucasians, potentially due to genetic polymorphisms influencing bone remodeling pathways, such as variations in the LRP5 or VDR genes linked to bone density regulation [21–23]. Additionally, racial differences in fat distribution (visceral vs. subcutaneous adipose tissue) and muscle mass may modulate mechanical loading effects on bone. Hormonal factors, such as vitamin D status (influenced by skin pigmentation and sun exposure) may further contribute [24, 25]. Socioeconomic factors could also play a role in these disparities [26]. Future studies incorporating bioimpedance analysis or dual-energy X-ray absorptiometry (DXA)-derived fat/lean mass measurements are warranted to elucidate these mechanisms.

In subgroup analyses stratified by BMI tertiles, the positive association between BMI and lumbar BMD was observed exclusively in the highest tertile (Q3: BMI ≥ 40 kg/m²). Employing further threshold effect analysis, our findings revealed a U-shaped relationship between BMI and BMD, with a point of inflection at 36.1 kg/m^2^. When a BMI was less than 36.1 kg/m^2^, the relationship between BMI and lumbar BMD was negatively correlated. When a BMI was greater than 36.1 kg/m^2^, the relationship was positively correlated. The non-linear association likely reflects competing mechanisms influenced by the severity of obesity, as hereby explained. (1) *Metabolic dominance at lower obesity levels (BMI 30–36.1 kg/m²)*, as follows. In individuals with moderate obesity, metabolic dysfunction—driven by visceral adipose tissue accumulation—may suppress bone formation. Excess visceral fat secretes proinflammatory cytokines (TNF-α, IL-6) and adipokines (leptin), which promote oxidative stress and insulin resistance [10, 12]. These factors inhibit osteoblast differentiation and activity while stimulating osteoclastogenesis, leading to reduced BMD [10, 12]. Concurrently, insulin resistance impairs the anabolic effects of insulin-like growth factor 1 (IGF-1) on bone remodeling [8], supporting the observed inverse relationship between BMI and BMD. (2) *Mechanical and hormonal adaptation at higher obesity levels (BMI > 36.1 kg/m²)*, as follows. Beyond the inflection point, mechanical loading from increased body weight may override metabolic harms. We acknowledge BMI’s limitations in assessing tissue-specific effects and therefore advocate for future mechanistic studies incorporating dual-energy X-ray absorptiometry (DXA). Additionally, according to Wolff’s law, repetitive stress on the skeleton stimulates osteoblast-mediated bone formation to adapt to heavier loads [7]. Severe obesity is associated with elevated estrogen levels due to aromatization of androgens in subcutaneous adipose tissue [9], which exerts antiresorptive effects on bone. Furthermore, higher muscle mass relative to fat mass in some individuals with severe obesity could amplify mechanical benefits, as lean mass strongly correlates with BMD [27]. These adaptive responses may explain the positive correlation between BMI and BMD in the Q3 group (BMI > 40 kg/m²). The inflection point (BMI 36.1 kg/m²) likely marks a shift in the dominant regulatory mechanism. Below this point, metabolic dysfunction (inflammation and insulin resistance) outweighs mechanical stimuli. Above it, mechanical loading and compensatory hormonal changes (e.g., estrogen and IGF-1) become predominant. In addition, Song’s research also shows a positive correlation between BMI and lumbar spine BMD [28]. This dual-phase mechanism aligns with the “obesity paradox” where severe obesity may confer protective effects in certain contexts [29]. However, individual variability in fat distribution (visceral vs. subcutaneous) and muscle mass complicates this relationship, underscoring the need for body composition analysis in future studies.

Our study utilized a large, nationally representative sample from NHANES 2011–2020, enhancing the generalizability of our findings. The identification of a U-shaped relationship between BMI and lumbar BMD provides novel insights into the complex interplay between obesity and bone health. This nonlinear association underscores the need to consider BMI thresholds in clinical assessments, particularly for individuals with BMI ≥ 36.1 kg/m² where mechanical loading may outweigh metabolic risks.

However, our study has several limitations. First, the cross-sectional design inevitably limited the drawing of an inference of a causal correlation between BMI and lumbar BMD among obese adults. More fundamental mechanistic research was needed to understand their particular mechanism. Second, while BMI is a practical obesity metric in epidemiological settings, it does not differentiate fat mass from lean mass, which are key determinants of bone health. Although NHANES lacks direct body composition data (e.g., DXA-derived fat/lean mass), we adjusted for biochemical markers (albumin and creatinine) as surrogates. Hormonal profiles (e.g., IGF-1 and leptin) were also unavailable, potentially confounding the BMI-BMD relationship. Nevertheless, our findings highlight the need for future studies integrating body composition and hormonal assays to clarify whether the U-shaped association reflects mechanical adaptation or metabolic interplay. Although BMD remains a validated predictor of fracture risk in the general population [30], its utility may be attenuated in obese populations due to the dual regulatory effects of adipose tissue on bone metabolism [5]. For instance, Compston demonstrated that overweight/obese women exhibit higher lumbar BMD without proportional reduction in non-vertebral fracture risk, underscoring the need to integrate bone microarchitecture (trabecular bone score and TBS) or fall risk factors for comprehensive assessment [31]. The BMD rebound observed at BMI > 36.1 kg/m² in our study may reflect mechanoadaptive compensation, although its translation to fracture risk reduction requires prospective validation. These limitations do not invalidate the observed threshold effect but contextualize its interpretation.

## Conclusion

This study highlights a U-shaped association between BMI and lumbar BMD in obese adults, with a critical inflection point at 36.1 kg/m², while BMI ≥ 36.1 kg/m² may indicate protective mechanical effects on bone. Clinicians should interpret BMI within the broader context of body composition, hormonal profiles, and lifestyle factors. We emphasize that BMI should serve as a complementary tool—not a replacement—for detailed assessments of bone health, particularly in obese populations. Further research integrating body composition analysis is needed to clarify the mechanisms underlying this nonlinear relationship.

## Electronic supplementary material

Below is the link to the electronic supplementary material.

Supplementary Material 1

## Data Availability

All data generated or analyzed during this study are included in this article. Further enquiries may be directed to the corresponding author.

## Abbreviations

BMI: Body mass index
EL: Education level
AL: Albumin
ALT: Alanine aminotransferase
AST: Aspartate aminotransferase
ALP: Alkaline phosphatase
BUN: Blood urea nitrogen
CAL: Total calcium
CPK: Creatine phosphokinase
CR: Creatinine
GGT: Gamma glutamyl transferase
GLU: Glucose
IR: Iron
PHO: Phosphorus
BIL: Total bilirubin
PRO: Total protein
UA: Uric acid
SOD: Sodium
POT: Potassium
CL: Chloride
OSM: Osmolality
GLO: Globulin
BMD: Total spine BMD
