# Supplementary material for: The association between body mass index and total lumbar bone mineral density in obese adults: the national health and nutrition examination survey (NHANES) 2011–2020
Source: Hormones (Athens). 2025 Jul 24;24(4):971–9. doi: 10.1007/s42000-025-00699-3 (PMC12678456; doi:10.1007/s42000-025-00699-3)
Supplement: Supplementary file 1 — Supplementary Material 1 [file 42000_2025_699_MOESM1_ESM.docx]

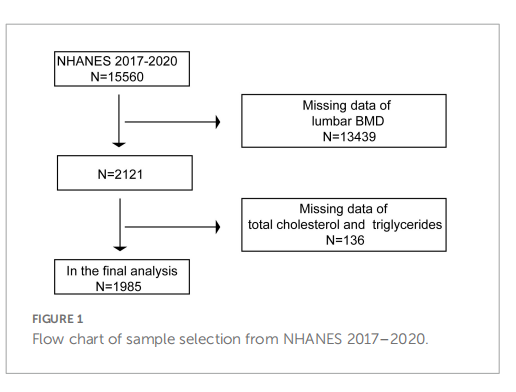
FLOWCHART

45462 TOTAL

31640 BMD MISS

13822LEFT

43 bmi miss

13779 left

2265+1+4+4+10+11+9+7xiebianliang miss

2311miss

11468 left

1541 less than 17sui

9927 left

3016 BMI<25+3203<30

3708 left

BMI

18.5 18.5-24.9 》=25 25-29.9 30-34.9 35-39.9 》=40

体重过低 正常 超重 肥胖前期 肥胖 重度肥胖 极重度肥胖

18.5 24.9 29.9

**研究人群描述**

|  | <35 | >=35, <40 | >=40 | P value |
| --- | --- | --- | --- | --- |
| AGE | 46.267 ± 13.660 | 45.679 ± 13.873 | 44.671 ± 12.746 | 0.02688 |
| ALBUMIN | 42.808 ± 3.185 | 41.785 ± 3.240 | 40.529 ± 3.237 | <0.00001 |
| ALT | 29.737 ± 19.825 | 29.096 ± 20.719 | 29.325 ± 20.731 | 0.70021 |
| AST | 26.419 ± 13.918 | 25.507 ± 22.302 | 25.803 ± 13.953 | 0.32995 |
| ALP | 70.029 ± 23.951 | 72.667 ± 22.605 | 75.835 ± 28.336 | <0.00001 |
| BUN | 4.792 ± 1.615 | 4.786 ± 1.943 | 4.693 ± 2.128 | 0.44356 |
| CALCIUM | 2.340 ± 0.085 | 2.330 ± 0.093 | 2.316 ± 0.087 | <0.00001 |
| CKP | 158.245 ± 146.828 | 151.529 ± 128.562 | 153.510 ± 171.640 | 0.46679 |
| CREATININE | 77.251 ± 24.694 | 75.968 ± 30.365 | 74.908 ± 31.776 | 0.12606 |
| GGT | 34.709 ± 44.656 | 31.752 ± 43.549 | 30.549 ± 31.368 | 0.04021 |
| GLUCOSE | 5.687 ± 2.050 | 6.090 ± 2.442 | 6.253 ± 2.495 | <0.00001 |
| IRON | 15.238 ± 6.247 | 13.530 ± 5.181 | 12.285 ± 5.544 | <0.00001 |
| PHOSPHORUS | 1.194 ± 0.174 | 1.162 ± 0.179 | 1.189 ± 0.182 | 0.00002 |
| BILIRUBIN | 10.195 ± 4.801 | 9.392 ± 5.501 | 8.717 ± 4.950 | <0.00001 |
| TOTALPROTEIN | 71.054 ± 4.296 | 70.635 ± 4.539 | 70.840 ± 4.422 | 0.04666 |
| URICACID | 5.659 ± 1.338 | 5.793 ± 1.392 | 6.012 ± 1.460 | <0.00001 |
| SODIUM | 139.108 ± 2.306 | 139.066 ± 2.313 | 138.876 ± 2.506 | 0.08081 |
| POTASSIUM | 3.985 ± 0.319 | 3.966 ± 0.321 | 4.012 ± 0.341 | 0.01858 |
| CHLORIDE | 103.658 ± 2.915 | 103.640 ± 3.058 | 103.517 ± 3.077 | 0.56027 |
| OSMOLALITY | 278.059 ± 4.960 | 278.374 ± 5.271 | 278.045 ± 5.186 | 0.25365 |
| GLOBULIN | 28.246 ± 4.099 | 28.851 ± 4.413 | 30.311 ± 4.167 | <0.00001 |
| LUMBAR.SPINE.BMD | 1.039 ± 0.152 | 1.036 ± 0.155 | 1.089 ± 0.158 | <0.00001 |
| GENDER |  |  |  | <0.00001 |
| 1 | 54.117 | 43.321 | 35.197 |  |
| 2 | 45.883 | 56.679 | 64.803 |  |
| RACE |  |  |  | 0.00002 |
| 1 | 63.119 | 60.177 | 57.897 |  |
| 2 | 12.494 | 16.397 | 20.837 |  |
| 3 | 11.438 | 11.813 | 11.037 |  |
| 4 | 12.950 | 11.613 | 10.230 |  |
| EDUCATION.LEVEL |  |  |  | 0.34385 |
| 1 | 38.367 | 36.665 | 34.995 |  |
| 2 | 60.193 | 61.353 | 62.824 |  |
| 3 | 1.440 | 1.982 | 2.181 |  |

Mean +/- SD for: AGE ALBUMIN ALT AST ALP BUN CALCIUM CKP CREATININE GGT GLUCOSE IRON PHOSPHORUS BILIRUBIN TOTALPROTEIN URICACID SODIUM POTASSIUM CHLORIDE OSMOLALITY GLOBULIN LUMBAR.SPINE.BMD . P value was calculated by weighted linear regression model.
% for: GENDER RACE EDUCATION.LEVEL . P value was calculated by weighted chi-square test.
Created by EmpowerStats (www.empowerstats.com) and R on 2022-03-29

**研究人群描述**

|  | <35 | >=35, <40 | >=40 | P value |
| --- | --- | --- | --- | --- |
| AGE | 46.27 ± 13.66 | 45.68 ± 13.87 | 44.67 ± 12.75 | 0.0269 |
| ALBUMIN | 42.81 ± 3.18 | 41.78 ± 3.24 | 40.53 ± 3.24 | <0.0001 |
| ALT | 29.74 ± 19.82 | 29.10 ± 20.72 | 29.33 ± 20.73 | 0.7002 |
| AST | 26.42 ± 13.92 | 25.51 ± 22.30 | 25.80 ± 13.95 | 0.3299 |
| ALP | 70.03 ± 23.95 | 72.67 ± 22.61 | 75.84 ± 28.34 | <0.0001 |
| BUN | 4.79 ± 1.61 | 4.79 ± 1.94 | 4.69 ± 2.13 | 0.4436 |
| CALCIUM | 2.34 ± 0.08 | 2.33 ± 0.09 | 2.32 ± 0.09 | <0.0001 |
| CKP | 158.25 ± 146.83 | 151.53 ± 128.56 | 153.51 ± 171.64 | 0.4668 |
| CREATININE | 77.25 ± 24.69 | 75.97 ± 30.37 | 74.91 ± 31.78 | 0.1261 |
| GGT | 34.71 ± 44.66 | 31.75 ± 43.55 | 30.55 ± 31.37 | 0.0402 |
| GLUCOSE | 5.69 ± 2.05 | 6.09 ± 2.44 | 6.25 ± 2.49 | <0.0001 |
| IRON | 15.24 ± 6.25 | 13.53 ± 5.18 | 12.28 ± 5.54 | <0.0001 |
| PHOSPHORUS | 1.19 ± 0.17 | 1.16 ± 0.18 | 1.19 ± 0.18 | <0.0001 |
| BILIRUBIN | 10.19 ± 4.80 | 9.39 ± 5.50 | 8.72 ± 4.95 | <0.0001 |
| TOTALPROTEIN | 71.05 ± 4.30 | 70.64 ± 4.54 | 70.84 ± 4.42 | 0.0467 |
| URICACID | 5.66 ± 1.34 | 5.79 ± 1.39 | 6.01 ± 1.46 | <0.0001 |
| SODIUM | 139.11 ± 2.31 | 139.07 ± 2.31 | 138.88 ± 2.51 | 0.0808 |
| POTASSIUM | 3.98 ± 0.32 | 3.97 ± 0.32 | 4.01 ± 0.34 | 0.0186 |
| CHLORIDE | 103.66 ± 2.92 | 103.64 ± 3.06 | 103.52 ± 3.08 | 0.5603 |
| OSMOLALITY | 278.06 ± 4.96 | 278.37 ± 5.27 | 278.05 ± 5.19 | 0.2536 |
| GLOBULIN | 28.25 ± 4.10 | 28.85 ± 4.41 | 30.31 ± 4.17 | <0.0001 |
| LUMBAR.SPINE.BMD | 1.04 ± 0.15 | 1.04 ± 0.16 | 1.09 ± 0.16 | <0.0001 |
| GENDER |  |  |  | <0.0001 |
| 1 | 54.12 | 43.32 | 35.20 |  |
| 2 | 45.88 | 56.68 | 64.80 |  |
| RACE |  |  |  | <0.0001 |
| 1 | 63.12 | 60.18 | 57.90 |  |
| 2 | 12.49 | 16.40 | 20.84 |  |
| 3 | 11.44 | 11.81 | 11.04 |  |
| 4 | 12.95 | 11.61 | 10.23 |  |
| EDUCATION.LEVEL |  |  |  | 0.3439 |
| 1 | 38.37 | 36.67 | 35.00 |  |
| 2 | 60.19 | 61.35 | 62.82 |  |
| 3 | 1.44 | 1.98 | 2.18 |  |

Mean +/- SD for: AGE ALBUMIN ALT AST ALP BUN CALCIUM CKP CREATININE GGT GLUCOSE IRON PHOSPHORUS BILIRUBIN TOTALPROTEIN URICACID SODIUM POTASSIUM CHLORIDE OSMOLALITY GLOBULIN LUMBAR.SPINE.BMD . P value was calculated by weighted linear regression model.
% for: GENDER RACE EDUCATION.LEVEL . P value was calculated by weighted chi-square test.
Created by EmpowerStats (www.empowerstats.com) and R on 2022-03-29

**多个回归方程**

| Exposure | Non-adjusted | Adjust I | Adjust II |
| --- | --- | --- | --- |
| BMI | 0.003 (0.002, 0.004) <0.00001 | 0.003 (0.002, 0.004) <0.00001 | 0.003 (0.002, 0.004) <0.00001 |

表中数据： β (95%CI) Pvalue / OR (95%CI) Pvalue
结果变量: LUMBAR.SPINE.BMD
暴露变量: BMI
Non-adjusted model adjust for: None
Adjust I model adjust for: GENDER; AGE; RACE
Adjust II model adjust for: GENDER; AGE; RACE; EDUCATION.LEVEL; ALBUMIN; ALT; AST; ALP; BUN; CALCIUM; CKP; CREATININE; GGT; GLUCOSE; IRON; PHOSPHORUS; BILIRUBIN; TOTALPROTEIN; URICACID; SODIUM; POTASSIUM; CHLORIDE; OSMOLALITY; GLOBULIN
此表用易侕统计软件 (www.empowerstats.com) 和R软件生成，生成日期： 2022-03-29

各模型所用的样本量

| Outcome | Exposure | Non-adjusted | Adjust I | Adjust II |
| --- | --- | --- | --- | --- |
| LUMBAR.SPINE.BMD | BMI | 3708 | 3708 | 3708 |

**多个回归方程**

| Model | GENDER= 1 | GENDER= 2 | Total |
| --- | --- | --- | --- |
| Non-adjusted | 0.005 (0.003, 0.006) <0.00001 | 0.003 (0.002, 0.004) <0.00001 | 0.004 (0.003, 0.004) <0.00001 |
| Adjust I | 0.004 (0.003, 0.006) <0.00001 | 0.002 (0.001, 0.003) 0.00002 | 0.003 (0.002, 0.004) <0.00001 |
| Adjust II | 0.004 (0.003, 0.006) <0.00001 | 0.002 (0.001, 0.003) 0.00017 | 0.003 (0.002, 0.004) <0.00001 |

表中数据： β (95%CI) Pvalue / OR (95%CI) Pvalue
结果变量: LUMBAR.SPINE.BMD
暴露变量: BMI
Non-adjusted model adjust for: None
Adjust I model adjust for: AGE; RACE
Adjust II model adjust for: AGE; RACE; EDUCATION.LEVEL; ALBUMIN; ALT; AST; ALP; BUN; CALCIUM; CKP; CREATININE; GGT; GLUCOSE; IRON; PHOSPHORUS; BILIRUBIN; TOTALPROTEIN; URICACID; SODIUM; POTASSIUM; CHLORIDE; OSMOLALITY; GLOBULIN
此表用易侕统计软件 (www.empowerstats.com) 和R软件生成，生成日期： 2022-03-29

各模型所用的样本量

| Outcome | Model | Exposure | GENDER= 1 | GENDER= 2 | Total |
| --- | --- | --- | --- | --- | --- |
| LUMBAR.SPINE.BMD | Non-adjusted | BMI | 1610 | 2098 | 3708 |
| LUMBAR.SPINE.BMD | Adjust I | BMI | 1610 | 2098 | 3708 |
| LUMBAR.SPINE.BMD | Adjust II | BMI | 1610 | 2098 | 3708 |

**多个回归方程**

| Model | RACE= 1 | RACE= 2 | RACE= 3 | RACE= 4 | Total |
| --- | --- | --- | --- | --- | --- |
| Non-adjusted | 0.003 (0.001, 0.004) 0.00159 | 0.003 (0.001, 0.005) 0.00025 | 0.004 (0.002, 0.006) 0.00010 | 0.003 (0.001, 0.005) 0.00162 | 0.003 (0.002, 0.004) <0.00001 |
| Adjust I | 0.003 (0.001, 0.005) 0.00062 | 0.003 (0.002, 0.005) 0.00003 | 0.004 (0.002, 0.006) 0.00004 | 0.003 (0.001, 0.005) 0.00102 | 0.003 (0.002, 0.004) <0.00001 |
| Adjust II | 0.003 (0.001, 0.004) 0.00379 | 0.003 (0.001, 0.005) 0.00049 | 0.004 (0.002, 0.007) 0.00003 | 0.003 (0.001, 0.005) 0.00895 | 0.003 (0.002, 0.004) <0.00001 |

表中数据： β (95%CI) Pvalue / OR (95%CI) Pvalue
结果变量: LUMBAR.SPINE.BMD
暴露变量: BMI
Non-adjusted model adjust for: None
Adjust I model adjust for: AGE; GENDER
Adjust II model adjust for: AGE; GENDER; EDUCATION.LEVEL; ALBUMIN; ALT; AST; ALP; BUN; CALCIUM; CKP; CREATININE; GGT; GLUCOSE; IRON; PHOSPHORUS; BILIRUBIN; TOTALPROTEIN; URICACID; SODIUM; POTASSIUM; CHLORIDE; OSMOLALITY; GLOBULIN
此表用易侕统计软件 (www.empowerstats.com) 和R软件生成，生成日期： 2022-03-29

各模型所用的样本量

| Outcome | Model | Exposure | RACE= 1 | RACE= 2 | RACE= 3 | RACE= 4 | Total |
| --- | --- | --- | --- | --- | --- | --- | --- |
| LUMBAR.SPINE.BMD | Non-adjusted | BMI | 1241 | 1106 | 658 | 703 | 3708 |
| LUMBAR.SPINE.BMD | Adjust I | BMI | 1241 | 1106 | 658 | 703 | 3708 |
| LUMBAR.SPINE.BMD | Adjust II | BMI | 1241 | 1106 | 658 | 703 | 3708 |

**单因素分析**

|  | Statistics | LUMBAR.SPINE.BMD |
| --- | --- | --- |
| BMI 分组 |  |  |
| <35 | 2020 (54.477%) | 1.045 (1.038, 1.051) |
| >=35, <40 | 965 (26.025%) | 1.041 (1.031, 1.051) |
| >=40 | 723 (19.498%) | 1.094 (1.083, 1.106) *** |
| P trend |  | <0.001 |

表中数据：adjust Mean (95% CI)
结果变量: LUMBAR.SPINE.BMD
暴露变量: BMI 分组
调整变量: None
此表用易侕统计软件 (www.empowerstats.com) 和R软件生成，生成日期： 2022-03-29

**单因素分析**

|  | Statistics | LUMBAR.SPINE.BMD |
| --- | --- | --- |
| BMI 分组 |  |  |
| <35 | 2020 (54.477%) | 1.046 (1.038, 1.053) |
| >=35, <40 | 965 (26.025%) | 1.041 (1.031, 1.051) |
| >=40 | 723 (19.498%) | 1.092 (1.080, 1.104) *** |
| P trend |  | <0.001 |

表中数据：adjust Mean (95% CI)
结果变量: LUMBAR.SPINE.BMD
暴露变量: BMI 分组
调整变量: GENDER; AGE; RACE
此表用易侕统计软件 (www.empowerstats.com) 和R软件生成，生成日期： 2022-03-29

**单因素分析**

|  | Statistics | LUMBAR.SPINE.BMD |
| --- | --- | --- |
| BMI 分组 |  |  |
| <35 | 2020 (54.477%) | 1.047 (1.040, 1.055) |
| >=35, <40 | 965 (26.025%) | 1.039 (1.030, 1.049) |
| >=40 | 723 (19.498%) | 1.089 (1.078, 1.101) *** |
| P trend |  | <0.001 |

表中数据：adjust Mean (95% CI)
结果变量: LUMBAR.SPINE.BMD
暴露变量: BMI 分组
调整变量: GENDER; AGE; RACE; EDUCATION.LEVEL; ALBUMIN; ALT; AST; ALP; BUN; CALCIUM; CKP; CREATININE; GGT; GLUCOSE; IRON; PHOSPHORUS; BILIRUBIN; TOTALPROTEIN; URICACID; SODIUM; POTASSIUM; CHLORIDE; OSMOLALITY; GLOBULIN
此表用易侕统计软件 (www.empowerstats.com) 和R软件生成，生成日期： 2022-03-29

平滑曲线拟合

**阈值效应分析**

For exposure: BMI

| Outcome: | LUMBAR.SPINE.BMD |
| --- | --- |
| 模型 I |  |
| 一条直线效应 | 0.003 (0.002, 0.004) <0.0001 |
| 模型 II |  |
| 折点(K) | 36.1 |
| < K 段效应 1 | -0.003 (-0.006, -0.001) 0.0187 |
| > K 段效应 2 | 0.006 (0.004, 0.007) <0.0001 |
| 2与1的效应差 | 0.009 (0.005, 0.012) <0.0001 |
| 折点处方程预测值 | 1.029 (1.020, 1.038) |
| 对数似然比检验 | <0.001 |

表中数据： β (95%CI) Pvalue / OR (95%CI) Pvalue
结果变量: LUMBAR.SPINE.BMD
暴露变量: BMI
调整变量: GENDER; AGE; RACE; EDUCATION.LEVEL; ALBUMIN; ALT; AST; ALP; BUN; CALCIUM; CKP; CREATININE; GGT; GLUCOSE; IRON; PHOSPHORUS; BILIRUBIN; TOTALPROTEIN; URICACID; SODIUM; POTASSIUM; CHLORIDE; OSMOLALITY; GLOBULIN
此表用易侕统计软件 (www.empowerstats.com) 和R软件生成，生成日期： 2022-03-29

各模型所用的样本量

| Outcome | Exposure | N |
| --- | --- | --- |
| LUMBAR.SPINE.BMD | BMI | 3708 |
